# Autism Spectrum Disorders in forensic psychiatric investigations–patterns of comorbidity and criminality

**DOI:** 10.3389/fpsyt.2023.1168572

**Published:** 2023-08-09

**Authors:** Björn Hofvander, Thomas Nilsson, Ola Ståhlberg, Emma Claesdotter, Patricia Moberg, Klara Ahlbäck, Malin Hildebrand Karlén

**Affiliations:** ^1^Lund Clinical Research on Externalizing and Developmental Psychopathology, Department of Clinical Sciences Lund, Lund University, Lund, Sweden; ^2^Department of Forensic Psychiatry, Region Skåne, Trelleborg, Sweden; ^3^Centre of Ethics, Law and Mental Health, Department of Psychiatry and Neurochemistry, University of Gothenburg, Gothenburg, Sweden

**Keywords:** Autism Spectrum Disorders, crime, criminal responsibility, forensic psychiatric investigation, clinical subgroups

## Abstract

**Background:**

There are contradictory research findings regarding whether individuals with Autism Spectrum Disorders (ASDs) are more or less likely to commit crimes. The aims of the current study were to: (1) Describe psychiatric and crime-related characteristics of a large group of offenders with ASD who had undergone a Forensic Psychiatric Investigation (FPI). (2) Identify clinical subgroups among this group of offenders. (3) Investigate associations between the identified clinical subgroups and (a) psychiatric comorbidity (b) types of crimes and (c) criminal responsibility.

**Methods:**

The study cohort consists of all subjects (*n* = 831) who received an ASD-diagnosis at an FPI between 2002 and 2018 in Sweden. Descriptive and clinical, as well as crime related variables were obtained from the FPIs. Non-parametric (Pearson χ^2^, Fisher's exact and Mann-Whitney *U*-test) inferential statistics were used for analyses of between-group differences and effect sizes were reported. A Latent Class Analysis was used to identify homogeneous subgroups (or classes) from categorical characteristics.

**Results:**

The cohort consisted of 708 men and 123 women, aged 18 to 74 yrs. Two-thirds (66.7%) of the cohort had at least one other psychiatric diagnosis, the most prevalent was substance use disorder (SUD). A severe mental disorder, equivalent to lack of criminal responsibility, was most often reported among offenders with a comorbid diagnosis of schizophrenia spectrum disorder. The most common type of crime was violent crime. Three person-oriented clinical subgroups were identified; (1) ASD with few other diagnoses; (2) ASD and very high levels of SUDs, plus moderate levels of other externalizing disorders and psychotic psychopathology and (3) ASD and moderate to high levels of personality disorders (other than ASPD) and SUDs.

**Conclusion:**

Our results highlight the importance of all parts of the CJS to be prepared to handle offenders with ASD, often with high levels of additional psychiatric problems. Traditional approaches in treatment or other psychosocial interventions for ASD may need to be adapted to at least three general clinical profiles– one with mainly neurodevelopmental problems, one with a spectrum of externalizing problems and one with complex personality related difficulties.

## 1. Introduction

Judging from population-based studies persons with Autism Spectrum Disorders (ASDs) appear as no more or less likely to come into contact with the Criminal Justice System (CJS) as compared to non-autistic people (1–3). This contrasts with another strand of research showing individuals with ASD to be clearly overrepresented in some parts of the CJS, in particular, prison and forensic psychiatric settings (4), though the field struggles with poor study quality, and small and biased samples (5). The reasons behind these contradictory results are unclear but of the utmost importance to understand in order to disentangle different pathways to criminal offending within this multifaceted group. Also, based on the apparent prevalence of persons with ASD in the CJS and forensic psychiatric care, more knowledge is required regarding their needs in the CJS as well as how interventions and preventive strategies should be organized to adequately respond to them (6).

ASD is an heterogeneous neurodevelopmental disorder affecting core aspects of developmental processes, presenting itself as early-onset difficulties in social communication and restricted, repetitive behaviors and interests (7). The cognitive disabilities constituting ASD (i.e. deficits in social cognition) can affect the individual's level of criminal responsibility and, in some legislations, the ability to be tried in a court of law (8, 9). The decision to determine these questions is often a complex task referred to forensic mental-health examiners by the court. Article 13 of the Convention on the rights of persons with disabilities (10) guarantees access to justice in the context of disability as a human right and lays concrete and binding duties on state parties. Most jurisdictions regulate parts of the CJS (i.e., from police proceedings up to convictions and sentencing) for vulnerable defendants like individuals with ASD (3, 11).

Studies have shown that offenders with ASD in prison settings share several characteristics with offenders without autism. They are more often males, of which many also meet criteria for ADHD, show early life signs of conduct problems, and suffer from substance abuse to a greater extent compared to non-criminal individuals (12, 13). Furthermore, offenders with ASD seem to be defined by more co-occurring psychiatric pathology (1), a delayed diagnosis of ASD (2), and experiences of victimization and social isolation (3), compared to individuals with ASD in general. These individuals commit various offenses, but there appears to be a high proportion of violent offenses, particularly arson and sexual offenses (14). Previous research has also highlighted the challenges that face treatment programs for imprisoned offenders with ASD (15, 16), if not adapted to their specific needs.

Forensic psychiatry provides treatment for persons with severe and disabling mental disorders who have committed a serious crime. Although we still know relatively little about ASD in forensic psychiatric settings, early studies [e.g. (17)] suggested a clear overrepresentation in forensic psychiatric care compared to what would be expected from prevalence figures. The clinical characteristics of this group have been in focus in later studies [e.g. (18)]. van Buitenen et al. (19) reported prevalence rates of co-occurring conditions in a large sample (*n* = 394) of mentally ill offenders with ASD, recruited in penitentiary institutions in the Netherlands, and found very high levels of comorbidity, with 79% meeting criteria for at least one other clinical disorder. Particularly SUDs, schizophrenia and neurodevelopmental disorders other than ASD were common in this group. They also reported strikingly low prevalences of anxiety disorders (1%) and depressive disorders (3%), while very high rates of these disorders have been found in ASD groups outside the forensic setting [e.g., (20)].

To date there have only been a handful of publications studying and describing individuals with ASD undergoing a Forensic Psychiatric Investigation (FPI, i.e., being assessed on the grounds of suspected unaccountability in connection with a criminal suspicion or charge). Possibly the earliest study of the prevalence of neurodevelopmental disorders in an FPI setting was conducted by Siponmaa et al. (21). In their reassessment of 126 young (aged 15 to 22 yrs) offenders who had been investigated at the Forensic Psychiatric Department in Stockholm from 1990 through 1995, they found 15% meeting the criteria for an ASD and a high rate of autistic traits in subjects not given an ASD diagnosis. They did not report co-existing disorders but found arson to be overrepresented among the offenders with ASD, compared to non-ASD offenders. Söderström et al. (22, 23) reported an ASD prevalence of 18% in a group of 100 consecutive offenders going through an FPI in Sweden between 1998 and 2001. They found associations between autistic traits, ADHD symptoms and psychopathic traits but did report co-existing disorders. Neither Sipponmaa et al. nor Söderström et al. reported whether forensic psychiatric care was recommended or not by the FPI team for this subgroup of offenders with ASD. More recently, a Norwegian study was published describing all offenders investigated by the Norwegian Board of Forensic Examination and diagnosed with ASD (*n* = 48) between 2000 and 2010 (24). The forensic reports entailed 41 men and seven women, with a mean age of 28.3 yrs. Co-occurring diagnoses were common and 83% were diagnosed with at least one additional psychiatric disorder and 33% were diagnosed with an intellectual disability (ID). Apart from ID, drug-related disorders were most common (19%), followed by ADHD (15%), personality disorders (15%) and psychotic disorders (13%). Twenty-one offenders (44%) had committed a violent crime and 12 (25%) a sexual crime. Vandalism including arson had been committed by eight offenders (17%). Taken together, results from the Helvershou et al. study indicated that comprehensive comorbidity profiles (interacting with the ASD-symptomatology) among offenders with ASD is the rule rather than the exception, as well as highlighting the need to adapt assessment procedures and treatment interventions in CJS and forensic psychiatric care to a broad range of psychiatric profiles for the ASD-group.

Based on the cited research and ASD as such, there is reason to believe that individuals with ASD who undergo an FPI constitute a particularly vulnerable group where more knowledge is urgently needed. Since there is no univocal evidence that ASD per se increases the risk of committing a crime it is imperative that we learn more about this group, what kind of criminal behavior brings them to court, what defines their psychiatric needs and how we appraise their criminal responsibility.

The aims of the current study were to:

1. Describe psychiatric and crime-related characteristics of a large group of offenders with ASD going through an FPI
2. Identify clinical subgroups among this group of offenders
3. Investigate associations between these identified clinical subgroups among offenders with ASD regarding (a) psychiatric comorbidity (b) types of crimes and (c) criminal responsibility

## 2. Methods

### 2.1. Participants

The study cohort consists of all subjects (*n* = 831) assigned with an ASD-diagnosis within an FPI between the years 2002 to 2018 in Sweden. The study was approved by the Swedish Ethical Review Authority (#2019-01994) and conducted in accordance with the declaration of Helsinki.

### 2.2. Forensic psychiatric investigations in Sweden

A court-ordered FPI is mandatory for a sanction to forensic psychiatric care in Sweden. Concerns about the defendant's mental health can be raised by the court before or during the trial. To receive forensic psychiatric care after sentencing, the offender must meet the medicolegal criteria for a severe mental disorder (SMD) and be found guilty of a crime severe enough to warrant a prison sentence. When the defendant is found guilty or confesses to the crime, an FPI can be commissioned by the court. Then, a team consisting of a forensic psychiatrist, a psychologist, a social worker and ward staff, assesses the defendant during the FPI and submits an expert report regarding the presence of an SMD to the court. The court then decides on the sanction.

### 2.3. Measures

#### 2.3.1. Procedures

All data included in the study originates from the central archive of the Forensic Medical Board in Sweden where finalized FPIs are stored and central information such as the conclusion of the FPI, diagnoses and crime are gathered in a digital registry. For the present study, information regarding the following variables from the registry was obtained via a record keeper with access to the central digital archive:

- Year of the FPI
- Age (yrs) at the time of FPI
- Sex (male/female)
- SMD at the time of the offense (yes/no)
- Diagnostic code(s) (DSM-IV-TR) axis I, II, III, IV and V
- Diagnostic code(s) (DSM-5)
- Crime code(s)

Before the coding of clinical diagnoses and crimes into categories, to be used within statistical analyses, data was de-identified by using code numbers. The same code number was used for all variables for each participant to ensure that data from all variables could be tied in patterns to each separate individual, which was necessary for conducting the Latent Class Analysis (LCA).

#### 2.3.2. Clinical diagnoses: content and coding

For the creation of codes, to be able to categorize psychiatric diagnoses, we had to consider that the sample's time period spanned over two versions of the DSM (DSM-IV and DSM-5).

##### 2.3.2.1. The ASD-diagnosis

The information regarding clinical diagnoses given in the FPIs was categorized according to DSM-IV from 2002 until 2015, and according to DSM-5 from 2016 until 2018. Regarding who was considered to have and ASD-diagnoses, the major DSM-IV subtypes of ASD (i.e. Autistic Disorder, Asperger's Disorder and Pervasive Developmental Disorder Not Otherwise Specified) were all categorized as ASD. One subject, who received a diagnosis of Childhood Disintegrative Disorder, was excluded due to previous research on the distinct nature of this rare syndrome (25). During the DSM-5 period, the ASD-diagnosis was used.

##### 2.3.2.2. Co-occurring psychiatric problems

Regarding which diagnoses/categories that were grouped together into respective code, see the full list below. The choices made regarding which diagnoses were grouped together into a code, were guided first of all, by the structure of major classes in DSM-5. Second, to avoid creating diagnostic groups with low *n*, we collapsed a number of individual diagnoses. The following diagnostic categories were created:

1. All subtypes of “Mental retardation”/“Intellectual disabilities” (ID) were pooled,
2. All subtypes of “Attention-Deficit/Hyperactivity Disorder” (ADHD), “Tic disorders” and “Conduct disorder” (CD) were pooled.
3. All other “Disorders first diagnosed in infancy, childhood, or adolescence”/“Neurodevelopmental disorders” were pooled and categorized as Other NDDs.

Diagnoses belonging to the following classes were also merged into separate diagnostic categories:

4. “Substance-related disorders” (excluding alcohol)/“Substance-related and addictive disorders” (excluding alcohol) – enabling the division of alcohol use disorder and other SUDs
5. “Schizophrenia and other psychotic disorders”/“Schizophrenia spectrum and other psychotic disorders”
6. “Depressive disorders” (in Mood disorders)
7. “Bipolar disorders” (in Mood disorder)
8. “Anxiety disorders” [excluding Obsessive-compulsive disorder and Posttraumatic stress disorder (PTSD)]
9. “Obsessive-compulsive disorder” (in Anxiety disorders) and “Trichotillomania” (in Impulse-control disorders not elsewhere classified)/“Obsessive-compulsive and related disorders”
10. “Paraphilias” except Pedophilia (in Sexual and gender identity disorders)/“Paraphilic disorders”
11. “Pedophilia” (in Sexual and gender identity disorders)/“Pedophilic disorder” (in Paraphilic disorders)
12. “Impulse-control disorders not elsewhere classified” (except Pathological gambling)/“Disruptive, impulse-control, and conduct disorders” (except Conduct disorder and Antisocial personality disorder, categorized as Other impulse-control disorders)
13. “Antisocial personality disorder” (in Personality disorders)
14. “Personality disorders” (except Antisocial personality disorder, categorized as Other personality disorders)
15. “Adjustment disorders” and PTSD/“Trauma and stressor-related disorders”
16. All other diagnoses were pooled and reported as Other disorders.

#### 2.3.3. Criminal offenses: content and coding

The cohort included offenders who had committed violent as well as non-violent crimes. All crime categories include attempted and aggravated forms wherever applicable. *Violent* crime was defined as homicide, manslaughter, assault, robbery, threats, and/or violence against an officer, interference in a judicial matter, gross violation of integrity, unlawful coercion and threats, kidnapping, illegal confinement, arson, or extortion [closely following previous Swedish definitions of violent crime, see, e.g., Falk et al. (26) and Fazel et al. (27), with minor revisions in accordance with United Nations Office on Drugs and Crime (28)]. *Deadly violence* was defined as murder and voluntary or involuntary manslaughter. *Aggravated violence* was defined as aggravated assault, kidnapping, or aggravated robbery.

*Sex* crimes were not included in the general category of violent crime but assigned their own category. Seven other non-violent crime categories were created and coded as follows: *Theft, Vandalism, Traffic, Weapons-related (e.g. unlawful possession of a weapon), Drug-related, Fraud* and *Economic* offenses, and *Other* crimes (a full detailed report of crimes included in each category is available from the corresponding author upon reasonable request).

### 2.4. Data analyses

Data were analyzed using SPSS 27 (SPSS, Chicago, IL, USA) and Latent Gold 6.0 (Statistical Innovations Inc., Arlington, MA) software using two-tailed *p*-values. The level of significance was set at *p* < 0.05. Due to non-normal distributions, we chose non-parametric methods for our analyses. Pearson χ^2^ and Fisher's exact test were used for analyses of between-group differences for categorical data. Mann-Whitney U test was used for continuous data. Effect size was calculated using Phi (Φ). According to Cohen's model (29), an effect size of 0.20 can be considered as small, of 0.30 medium, and of 0.50 large when Phi is used.

An LCA was used to identify homogeneous subgroups (or classes) from categorical characteristics (here: psychiatric DSM diagnoses) by using the program Latent Gold (30). In this cohort of offenders who underwent a forensic psychiatric investigation, a number of co-occurring DSM diagnoses were selected and grouped into the following six main diagnostic categories due to a. their relevance for the forensic psychiatric field regarding for example treatment, and b. to avoid groups of small n; occurrence of a Severe Mental Illness (SMI, i.e., either a Schizophrenia spectrum disorder or a Bipolar disorder), an Antisocial personality disorder (ASPD, including CD), any other personality disorder, any form of SUD, Intellectual disability, and ADHD. These variables were used in an LCA analysis modeling one-class to four-class solutions. There is no single way to decide which model is the optimal one (31). Instead, this must be done by evaluating theoretical meaningfulness and model fitness, as indicated by commonly used fit statistics such as Bayesian Information Criteria (BIC), Akaike Information Criteria (AIC), and the Vuong-Lo-Mendell-Rubin (VLMR) adjusted likelihood test ratio (see Table 3).

## 3. Results

The cohort consisted of 708 men (85.2%) and 123 women (14.8%), their mean age was 29.9 yrs (18–74 yrs, SD = 10.3) at the time of the FPI. In Table 1, the diagnoses, apart from ASD, set at the FPI are reported. Co-occurring conditions were common, 66.7% of the cohort had at least one additional psychiatric diagnosis. Most common was SUD (26.0%), closely followed by schizophrenia spectrum disorder (16.1%), ID (15.8%) and ADHD (15.4%). A total of 553 (66.5%) were considered to suffer from an SMD at the time of the offense.

**Table 1:** Diagnoses (*n*, %) at the time of the FPI for the total group and for those with an SMD, where the latter are compared to those without an SMD.

	**Total group *n =* 831 (100%)**	**SMD at offense *n =* 553 (66.5%)**	**Φ**	***p*-level**
Any SUD	216 (26.0%)	129 (59.7%)	−0.086	0.013^*^
Other SUDs	135 (16.2%)	84 (62.2%)	−0.040	0.245
Alcohol use disorder	110 (13.2%)	69 (62.7%)	−0.032	0.362
Schizophrenia spectrum disorder	134 (16.1%)	131 (97.8%)	0.290	< 0.001^***^
Intellectual disability	131 (15.8%)	113 (86.3%)	0.181	< 0.001^***^
ADHD	128 (15.4%)	74 (57.8%)	−0.079	0.023^*^
Other personality disorders	57 (6.9%)	36 (63.2%)	−0.019	0.574
Conduct disorder	48 (5.8%)	24 (50%)	−0.087	0.012^*^
Other impulse-control disorders	47 (5.7%)	42 (89.4%)	0.118	< 0.001^***^
Depressive disorder	34 (4.1%)	22 (64.7%)	−0.008	0.816
Obsessive-compulsive disorders	32 (3.9%)	26 (81.3%)	0.062	0.072
Tic disorder	32 (3.9%)	25 (78.1%)	0.049	0.157
ASPD	31 (3.7%)	14 (45.2%)	−0.089	0.010^**^
Other disorders	23 (2.8%)	18 (78.3%)	0.042	0.227
Pedophilic disorder	21 (2.5%)	14 (66.7%)	0.000	0.991
Trauma and stress-related disorders	18 (2.2%)	7 (38.9%)	−0.087	0.012^*^
Anxiety disorder	17 (2.0%)	6 (35.3%)	−0.096	0.006^**^
Other paraphilias	15 (1.8%)	10 (66.7%)	0.000	0.992
Bipolar disorder	14 (1.7%)	12 (85.7%)	0.053	0.159^†^
Other neurodevelopmental disorders	13 (1.6%)	6 (46.2%)	−0.054	0.116
Any psychiatric comorbidity	554 (66.7%)	399 (72.0%)	0.164	< 0.001^***^

^†^Fisher's exact test.

^*^p = 0.05; ^**^p = 0.01; ^***^p < 0.001.

Female patients were significantly more often assessed as having an SMD at the time of the offense (79.7% compared to 64.3% of the men, *p* < 0.001, Φ = 0.116). Age was not related to whether the offender was considered to meet the requirements for an SMD at the time of the offense (Mann-Whitney *U* = 78,598.000, *p* = 0.596).

The crimes committed by the cohort are presented in Table 2. Violent crimes were dominant, followed by sexual offenses, vandalism and theft. Only a small minority (7.3%) was prosecuted for a drug-related crime.

**Table 2:** Types of criminal acts (*n*, %) in the prosecution for the total group and for those with an SMD, where the latter are compared to those without an SMD.

	**Total group *n =* 831 (100%)**	**SMD at offense *n =* 553 (66.5%)**	**Φ**	***p*-level**
Violence	627 (75.5%)	439 (70.0%)	0.129	< 0.001^***^
Aggravated violence	86 (10.3%)	53 (61.6%)	−0.035	0.307
Deadly violence	101 (12.2%)	64 (63.4%)	−0.025	0.470
Sexual	134 (16.1%)	70 (52.2%)	−0.133	< 0.001^***^
Vandalism	109 (13.1%)	79 (72.5%)	0.049	0.159
Theft	86 (10.3%)	54 (62.8%)	−0.027	0.436
Drug-related	61 (7.3%)	38 (62.3%)	−0.025	0.465
Fraud and economic	34 (4.1%)	21 (61.8%)	−0.021	0.546
Weapons-related	31 (3.7%)	17 (54.8%)	−0.049	0.159
Traffic	22 (2.6%)	13 (59.1%)	−0.026	0.453
Other crimes	219 (26.4%)	155 (70.8%)	0.054	0.122

^***^p < 0.001.

Table 3 presents data for fit statistics from a series of latent class models ranging from one to four classes for the cohort of ASD individuals who underwent a forensic psychiatric investigation. Among the fit statistics presented are the Bayesian Information Criterion (BIC; a goodness-of-fit index that considers the rule of parsimony), and the Akaike Information Criteria (AIC and AIC3; an estimator of prediction error that estimates the amount of information lost by the model). In addition to these the model fit tests of the L2 statistic, and the Vuong-Lo-Mendell-Rubin (VLMR) adjusted likelihood test ratio test are also presented. When evaluating these fit statistics against each other the three-way class solution was found to be the best one, since this model was supported by the AIC3 information criteria showing the best weighting between model fit and parsimony. This solution was also supported by an acceptable model fit according to L2 showing a greater *p*-value than for the two-way class solution and fewer parameters than the four-way class solution thus indicating this model as the most parsimonious one. Finally, the VLMR showed a higher degree of statistical significance compared to the latent four-class model (statistics in bold).

**Table 3:** Model fit statistics for LCA with 1–4 classes of comorbid DSM- diagnoses.

**Class**	**LL**	**BIC (LL)**	**AIC (LL)**	**AIC3 (LL)**	**NPAR**	**L^2^**	**df**	***p***	**VLMR**	***p***
1	−974.25	3,988.84	3,960.51	3,966.51	6	119.80	57	2,30E-06		
2	−946.06	3,979.51	3,918.12	3,931.12	13	63.40	50	0.10	56.39	0
3	−934.95	4,004.35	3,909.90	**3,929.90**	20	**41.19**	43	**0.55**	**22.21**	**0.0007**
4	−927.61	4,036.74	3,909.23	3,936.23	27	26.51	36	0.87	14.67	0.0525

The bold values indicate the best latent class model with the Akaike Information Criteria (AIC) as an estimator of prediction error, model fit tests of the L2 statistic, and the Vuong-Lo-Mendell-Rubin (VLMR) adjusted likelihood test ratio test.

**Figure 1:**
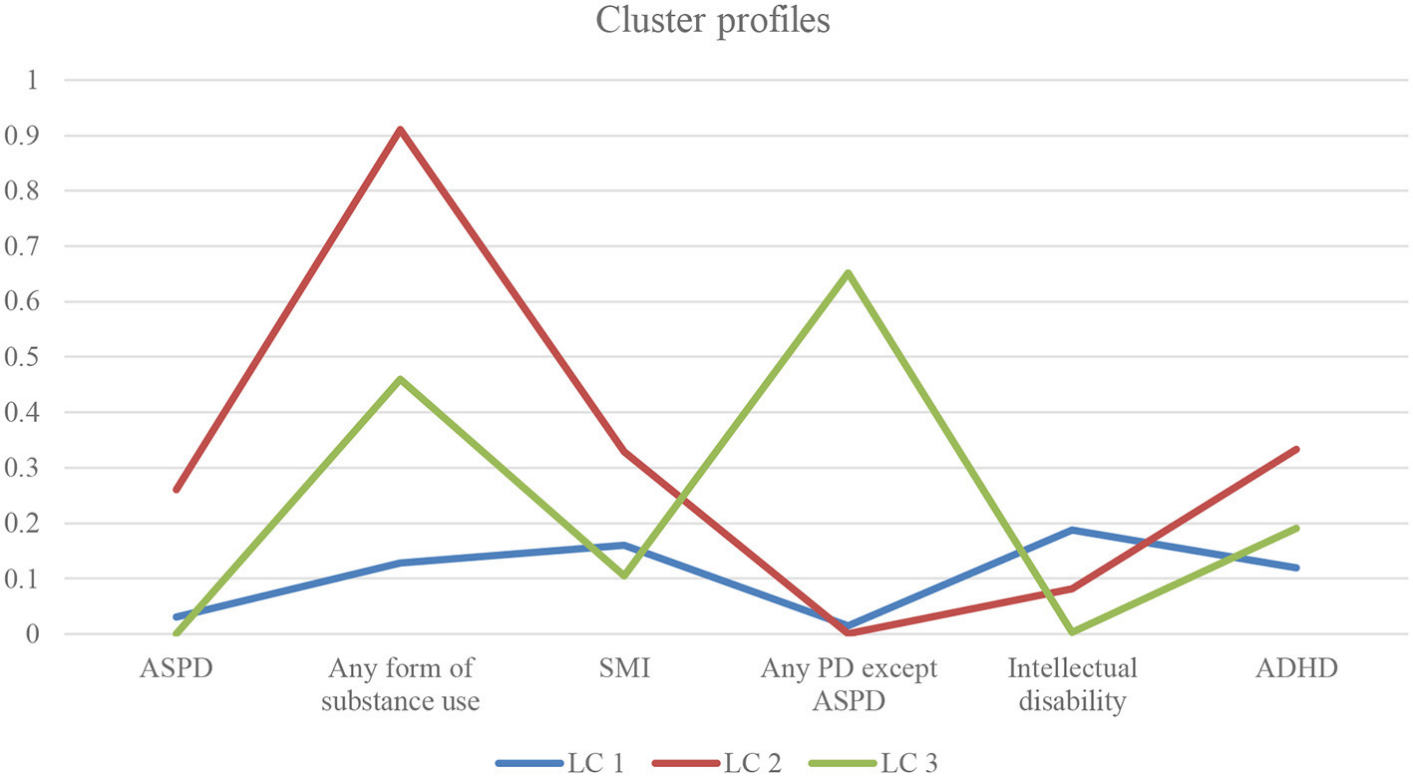
Proportion of individuals with selected co-occurring diagnoses for the three latent classes (LC). The curve depicts the proportion of each LC diagnosed with the included diagnostic group. ASPD, Antisocial Personality Disorder; SMI, Severe Mental Illness; PD, Personality Disorder; ADHD, Attention-Deficit/Hyperactivity Disorder.

Figure 1 presents the proportion of each LC that were diagnosed with each included diagnostic group. A qualitative inspection of the three latent class profiles revealed a theoretically interpretable and meaningful picture that further supports this three-way class solution. This model could be summarized as one large class (LC 1) consisting of individuals (*n* = 686) with almost no co-occurring diagnoses besides their ASD, though slightly higher levels of ID, compared to the other two groups. A second class (LC 2) entailing individuals (*n* = 90) characterized by high levels of SMI, an extremely high prevalence of SUDs, high ADHD and ASPD, and a third class (LC 3) containing individuals (*n* = 55) that to a quite large amount met criteria for a personality disorder other than ASPD, often in combination with some form of substance use (see Figure 1).

No LC-group was significantly more often considered to meet requirements for an SMD at the time of the offense (LC1, 67.3%, Φ = 0.037, *p* = 0.287; LC2, 63.3%, Φ = −0.024, *p* = 0.494; LC3, 61.8%, Φ = −0.027, *p* = 0.442).

## 4. Discussion

To summarize, we aimed to describe the psychiatric and crime-related characteristics of a large cohort of offenders with ASD going through an FPI, using variable-oriented and also person-oriented methods. The variable-oriented analyses showed that the three most common types of crimes among these offenders were violent crime, sexual crime or vandalism. Regarding psychiatric comorbidity, the most prevalent co-existing disorder was SUD, followed by schizophrenia spectrum disorder, ID, and ADHD. Not surprisingly, an SMD, i.e., equivalent to a lack of legal liability in many legal systems, was most often deemed among those offenders with a co-occurring diagnosis of schizophrenia spectrum disorder, but also impulse-control disorders (other than Conduct disorder and ASPD), ID and OCD. Among the larger clinical categories, Conduct disorder, ASPD, ADHD and SUD, i.e., externalizing disorders, were all negatively related to an SMD decision at the FPI. In the person-oriented analysis, three clinical subgroups (LC 1–3) within this cohort were identified: subgroup 1 was characterized by only ASD where ID also was seen in a minority of this group; subgroup 2 by, in addition to their ASD, high levels of psychiatric comorbidity, particularly within the externalizing spectrum where substance use stood out as occurring in almost every case; and subgroup 3 that was defined by personality disorders (other than ASPD) and often also a SUD.

### 4.1. Factors among offenders with ASD often associated with SMD

As expected from other studies of perpetrators of violent crimes (32, 33), the vast majority of these offenders (85.2%) were male. They were also rather young which is in line with the crime age curve, which states that criminal activity peaks before the age of 35 years (34). However, as seen in previous studies on subjects in FPIs, women with ASD undergoing an FPI were comparatively more often found to suffer from an SMD [e.g. (35)].

#### 4.1.1. Criminality

In terms of offenses, the most common crime category was violent crime, present in three quarters (75.5%) of the prosecutions. Sexual crimes were the second most common crime (16.1%). This is in line with previous studies [e.g. (36)] where different interpersonal crimes are dominant among offenders with ASD, but interpersonal crimes are also the most common among the offenders where an FPI is requested by the court. The results regarding type of crime showed that offenders with ASD were substantially more often considered to meet requirements for an SMD (than not to) when they had committed a violent crime (70.0%, compared to 66.5% in general). Vandalism was also a common crime among those with SMD, though it did not reach significance. This should be compared to sexual crimes where only ~50% of the offenders were considered to have an SMD when committing these types of crimes.

#### 4.1.2. Co-occurring psychiatric conditions

Psychiatric comorbidity was very common in this group, where two-thirds (66.7%) received at least one other psychiatric diagnosis at the FPI. A SUD was the most common additional diagnosis, affecting approximately a quarter of the group (26%). A previous, large study based on population registers (37) has found an increased risk of SUDs among individuals with ASD, and clinical studies have shown high prevalence figures as high as 19–30% among patients with an ASD diagnosis within general psychiatric care (20, 38). There is also a strong and well-documented relationship between SUDs and criminality and prevalence rates of these disorders are high in convicted samples [e.g. (39)]. It should be noted that within the present cohort, distribution was relatively even between SUD diagnoses related to alcohol and narcotics.

A relatively sizeable minority were also diagnosed with psychotic disorders and our results were similar (16.1 vs. 13%) to what was found in a small Norwegian FPI sample (24). However, these figures of psychotic co-occurring diagnoses at the FPI-stage are considerably lower than what has been found for patients with ASD treated within forensic psychiatric care (19). It is, in line with this, noteworthy that almost all of the offenders in the present cohort with a co-occurring psychotic diagnosis (97.8%) were considered to meet the criteria for an SMD at the offense.

It is well known that there is a great co-occurrence of neurodevelopmental disorders in those with ASD [e.g., (40)] and many of the subjects also met criteria for an IDs and/or ADHD, in addition to their ASD. The prevalence of ID was much higher in the Norwegian (24) sample (33% compared to our 15.8%), which is in line with a recent review of comorbidity between ASD and ID (41). The reasons behind this difference merits further investigation. Our ADHD levels were very much the same as the Norwegian, however (15.1 vs. 15%).

Just as van Buitenen et al. (19) reported very low rates of depression and anxiety, we also found surprisingly low prevalences (4.1% and 2.0%) of these diagnoses in the present cohort. Whether this indeed reflects a true picture, where ASD offenders present a low prevalence internalizing symptoms, future studies must tell. In addition, few offenders in this cohort received a diagnosis more directly related to a pattern of potentially criminal behavior, such as ASPD (3.7%) or Pedophilic disorder (2.5%).

### 4.2. The clinical subgroups and potential implications of these

A latent three-class model was found within the LCA to be superior to the other tested models. This model characterized three clinical subgroups among these offenders with ASD who had undergone an FPI. The largest clinical subgroup consisted of individuals with few other diagnoses than their ASD. The second-most prevalent clinical subgroup was characterized by very high levels of SUD, high levels of ADHD and ASPD and elevated levels of either a schizophrenia spectrum disorder or a bipolar disorder (i.e., an SMI). The third, and smallest, clinical subgroup contained individuals with personality disorders (other than ASPD) and, in many cases, also a SUD.

The clinical characteristics of the largest clinical subgroup, a group with “only” ASD (sometimes in combination with ID), have implications for the application of Swedish law as well as the subsequent treatment of and service provision for this offender group. Neurodevelopmental disorders, traditionally represented by ID, are not considered as an SMD according to Swedish law (42), but the “court may not impose a sentence of imprisonment if, as a result of the serious mental disturbance [SMD], the accused lacked the capacity to realize the implications of the act or to adapt their conduct accordingly” (43). Since the analyses showed that this group, subgroup 1, was not significantly overrepresented in either the SMD or the non-SMD groups, these clients are subsequently handled within both the prison and forensic psychiatric services. If legal practice has departed from the current legislation this warrants further analyses in future studies, where for example questions regarding reality testing and action control in offenders with ASD can be investigated.

The two remaining clinical subgroups were considerably smaller than the first. The second largest was characterized by offenders with ASD with SUD, but also other externalizing disorders [see HiTOP; (44)] as well as co-occurring psychotic psychopathology. These offenders with problems in multiple areas should be particularly difficult to assess within an FPI, due to the highly complex differential diagnostics considerations. This clinical subgroup seems heterogeneous in terms of SMD decisions by the FPI teams. While SMI (i.e. bipolar and schizophrenia spectrum disorders) are clearly related to SMD and approximately 60% of the offenders with ASD+SUD and ASD+ADHD problems (i.e., a majority) met criteria for an SMD, only 45% of the offenders with ASD+ASPD diagnosis did so (i.e., a minority in the SMD-group). From a treatment perspective, the combination ASD and externalizing disorders can be demanding to accommodate since, on the one hand, disruptive, norm-breaking and impulsive problems require clear boundaries and non-flexibility, while ASD, on the other hand, requires support, special education interventions and low-arousal methods to handle stressors. Facilitating and improving emotion regulation skills can however be a recommendable transdiagnostic intervention for this group.

For the last, and smallest, clinical subgroup, personality disorders (other than ASPD) and SUDs were found in addition to their ASD problems. Earlier studies have found that at least 50% of individuals with ASD meet the diagnostic criteria for at least one PD (20, 45). The intersection between ASD and personality disorders is an admittedly difficult clinical area (46), particularly in adults (47), and the lack of clinical guidance probably adds to these straits [e.g. (48)]. However, the high rate of known risk factors for a maladaptive personality development in forensic populations, such as childhood adversities (49) and ADHD [e.g. (50, 51)], may also predispose offenders with ASD to develop personality disorders. In addition, Keller et al. (52) recently suggested that delayed autism diagnosis and a lack of specific interventions at a young age are connected to a plethora of severe negative outcomes, including high rates of comorbidities and problem behaviors. Carthy and Murphy (48) emphasize the importance of accurate identification of personality disorders in offenders with ASD, since this poses opportunities for customized treatments as well as risk assessments.

Finally, we wanted to investigate associations between SMD, co-occurring conditions, the identified clinical subgroups and types of crimes. There were a number of characteristics significantly related to an offender with ASD being more likely to meet requirements for an SMD at the time of the offense, but a Schizophrenia spectrum disorder was the only clinical characteristic that had an effect size bordering to a moderate level (Φ = 0.290). All other variables were negligible in their relationship to an SMD at the time of the offense. Furthermore, no associations were found between any of the identified clinical subgroups and a higher prevalence of SMD. These results highlight the importance of forensic psychiatric care, as well as the CJS in general, being prepared to handle clients with ASD *in addition* to other severe psychiatric problems. Traditional approaches in treatment or other psychosocial interventions for ASD may need to be adapted to at least three general clinical profiles– one with mainly neurodevelopmental problems, one with a spectrum of externalizing problems and one with additive personality difficulties.

There are several limitations to this study. First the lack of a comparison group makes our results less valid. However, the cohort is consecutive and includes all FPI cases during the specific time period. Second, the time period included two different diagnostic manuals, the DSM-IV and DSM-5, which forced us to create aggregated clinical categories in a manner not intended in any of the manuals. Related to this, more specific diagnostic characterization could not be made within the clinical subgroups making it possible to investigate the clinical utility of these three clinical subgroups, especially why some in these respective groups are considered to meet requirements for an SMD and some are not, which should be the next step. Also, these subgroups could also be considered in the future from a rehabilitative perspective, how they fare within the forensic psychiatric care and the CJS, and to what extent each subgroup relapses in criminality and what crimes they then commit. A third limitation is the narrow demographic information (e.g. ethnicity, educational background and socio-economic data) available on the cohort. These data could provide valuable insights to our aims. Fourthly, the fact that Swedish law and practice guidelines emphasize that the FPI should be concluded when the court's question regarding if the severity of psychiatric problems are sufficient enough to meet requirements for an SMD, could result in a “comorbid blind spot” for this clinical subgroup. However, since ASD per se is not considered an SMD, this would be a minor problem, though future studies should examine this further.

To conclude, this study emphasizes the need of the CJS, as a whole, to be prepared to meet offenders with ASD, often with co-occurring psychiatric problems. Existing treatments and support models for individuals ASD may need to be adapted to at least three general clinical profiles– one with mainly neurodevelopmental problems, one with a spectrum of externalizing problems and one with complex personality related difficulties.

## Data availability statement

The raw data supporting the conclusions of this article will be made available by the authors, without undue reservation.

## Ethics statement

The studies involving human participants were reviewed and approved by Swedish Ethical Review Authority (#2019-01994). Written informed consent for participation was not required for this study in accordance with the national legislation and the institutional requirements.

## Author contributions

BH, TN, and MH conceived and designed the study. MH obtained the data. BH together with PM and KA controlled the data. BH and TN analyzed the data. BH drafted the initial manuscript. All authors critically revised the manuscript and approved of the final version.
